# Inequality as determinant of donation: A theoretical modeling and empirical analysis of Korea

**DOI:** 10.1371/journal.pone.0306370

**Published:** 2024-09-16

**Authors:** Sang Hak Sohn, Jong Mun Yoon, Seongmin Jeon

**Affiliations:** 1 PPS Company, Seoul, Korea; 2 The Credit Finance Association, The Credit Finance Research Institute, Seoul, Korea; 3 College of Business, Gachon University, Seongnam, Korea; Universita degli Studi del Piemonte Orientale Amedeo Avogadro, ITALY

## Abstract

Recipient financial need is a crucial factor in donation decisions. This study proposes a novel model for determining financial donations, incorporating consumption levels of both donor and recipient within a societal context. Solving our model’s utility maximization problem reveals how consumption, donation, and savings are interlinked. Empirical evidence reinforces these findings, aligning with prior research and showing that larger consumption gaps between donors and recipients lead to increased donations. Our findings point towards an inherent altruistic motivation in donation, where elevating the recipient’s well-being ultimately enhances the donor’s own utility. This reinforces the notion that consideration of the recipient’s financial hardship, as reflected by their consumption patterns, is crucial when making donation decisions. Shifting beyond traditional models, this study introduces a groundbreaking approach to financial donations. Our novel model factors in consumption levels of both the donor and recipient, along with the broader societal context, using utility maximization to unravel the intertwined decisions of consumption, donation, and savings. Real-world data validates our model, confirming known donation factors and revealing a key finding: larger disparities in consumption lead to increased giving, suggesting an altruistic drive where helping others boosts personal satisfaction.

## Introduction

This study challenges the assumption of pure self-interest by introducing a novel utility function that incorporates altruism. It shows how individuals, despite potential spending reductions, are motivated to donate, providing empirical evidence for their inherent generosity. While existing theoretical models explore why people donate, they often overlook the recipient’s consumption–a crucial factor influencing the impact and motivation for giving. This study bridges this gap by presenting a novel explanation for donation that incorporates both donor motives and recipient needs. Capitalizing on the stark contrast in marginal utility, the UNICEF campaign demonstrates how even small donations can significantly improve the lives of those in poverty. This effective strategy taps into our empathy and encourages giving by bridging the gap between donor and recipient. Moving beyond existing paradigms, this study analyzes donation through a novel economic utility lens. It proposes a groundbreaking model that incorporates both individual and societal consumption levels into the decision-making process of economic actors, revealing the recipient’s consumption as a key determinant of donation behavior. Moving beyond traditional models, this study pioneers the integration of recipient consumption into an individual’s utility function. This novel framework, encompassing both donor and recipient needs, redefines how we understand donation decisions by explicitly linking the act of giving to its impact on well-being. While individual income and other factors undoubtedly influence donation, existing research often overlooks the underlying mechanisms. This study dives deeper, exploring the decision-making process through the lens of individual utility. It posits that relative income and consumption play crucial roles, offering a novel framework for understanding why and how people donate. Beyond limitations of traditional models, this study unveils hidden levers of altruism by integrating relative consumption into a broader utility function. Armed with cutting-edge models and real-world data, we delve into the intricate dance of decision-making, revealing the true catalysts of individual giving. While European and American philanthropy are widely lauded, less is known about the burgeoning charitable spirit in South Korea. This study delves into the unique world of Korean giving, a relatively young phenomenon that emerged in the late 1980s, to uncover its underlying dynamics and shed light on its growing impact.

## Literature review

Building on the argument by Clark et al. (2008) [1] that relative income, not just absolute wealth, influences happiness and economic behavior, this study investigates how relative income and consumption affect donation decisions. By exploring these factors through the lens of individual utility, we aim to illuminate the underlying mechanisms driving donation behavior. This deeper understanding of donation motivations can potentially contribute to further insights into both happiness and economic choices. Donations are defined as a signal of generosity, motivated by the desire to appear generous and to receive social approval (Harbaugh, 1998 [2]). The theoretical and empirical literature has tried to identify the determinants of money donations and time volunteered. The literature is grouped into three categories: individual preferences and attitudes, charities behavior, and government behavior (Cappellari et al., 2011 [3]). Economists have begun to incorporate psychological factors into models of philanthropic behavior. This is because empirical evidence suggests that psychological factors play a role in explaining non-selfish behavior. Unveiling the multifaceted nature of giving, Benabou and Tirolle (2006) [4] proposes three distinct pathways to donor satisfaction: the inherent joy of generosity, the internal reward of a positive self-image, and the external validation of social respect. In exploring the economics of individual giving, three key models dominate: the public goods model, the private consumption model, and the impure public goods model. Each reveals distinct motivations behind donations, offering a nuanced understanding of why people give. Understanding why people donate hinges on three competing narratives. The public goods model sees individuals driven by a desire to improve society, while the private consumption model views giving as a source of personal satisfaction. Finally, the impure public goods model reconciles these perspectives, suggesting a blend of altruism and self-benefit motivates generosity. Table 1 below summarizes the discussed models.

**Table 1:** Individual donation models and utility functions.

	Utility Function	Notation
Public Goods Model	$U_{i}=U(C_{i},g_{i}+\sum_{j\neq i}g_{j})$	*C*_*i*_: Individual consumption, *g*_*i*_: Individual donation level,
		∑_*j*≠*i*_ *g*_*j*_: Sum of public goods excluding donation of the donator him/herself
Private Consumption Model	*U*_*i*_ = *U*(*C*_*i*_, *D*_*i*_)	*C*_*i*_: Individual consumption, *D*_*i*_: Donation level
Impure Public Goods Model	*U*_*i*_ = *U*(*C*_*i*_, *G*, *D*_*i*_)	*C*_*i*_: Individual consumption, *G*: Public goods level
		*D*_*i*_: Individual donation level

Son and Park (2008) [5] laid the groundwork with their insightful analysis of three key models: public goods, private consumption, and impure public goods. Their work offers fertile ground for further exploration, but also identifies potential blind spots. Before embarking on our own investigation, we would carefully examine these existing studies to glean their strengths and weaknesses, building a firmer foundation for our own understanding. While the public goods model predicts donations plummeting with increased government funding, reality paints a different picture, hinting at the model’s narrow view of giving behavior. Similarly, the private consumption model, focusing solely on personal satisfaction, neglects the potential societal impact of donations. Even the impure public goods model, though nuanced, assumes a binary choice between public good and personal satisfaction, limiting its explanatory power. A vast body of research delves into the factors influencing individual giving. Notably, studies consistently reveal a positive link between Christianity, particularly church attendance, and donation levels. Likewise, higher education and age emerge as reliable predictors of increased generosity. Delving deeper into individual characteristics, Wiepking and Bekkers (2012) [6] surveyed the landscape of research on gender, family, and income. While married couples tend to donate more, the link between children and giving remains nuanced, with some studies finding no clear positive association. In contrast, the connection between income and generosity receives strong backing from research institutions. Interestingly, the perceived visibility of the donation to the recipient also plays a role, with givers more likely to contribute when they feel their effort will resonate directly with the beneficiary. Moving beyond traditional models, this study pioneers the investigation of relative income and consumption as crucial determinants of donation. By focusing on these previously overlooked factors, it unveils a deeper understanding of the motivations and processes underlying charitable giving.

## Data and methods

Leveraging data from the 7^th^ (2004) to 20^th^ (2017) waves of the Korean Labor and Income Panel Study (KLIPS), a large-scale annual survey of 5,000 households and individuals in non-rural Korea, we delve into the factors influencing charitable giving. While the Korean Labor Panel provides valuable data, it only offers household-level consumption. To overcome this limitation, this study focuses solely on the household head. This enables us to align individual and household characteristics, ensuring consistency in our analysis of donation behavior. This study examines how relative income and relative consumption influence household charitable giving, measured as annual expenditure on donations. The KLIPS used the question to assess household contributions: "How much did your family spend on average per month on tithes and various donations throughout the past year?" Table 2 provides details of the data used in this analysis.

**Table 2:** Summary statistics.

Variables	Observations	Mean	SD	Min.	Max.
Engaged in Donation	81,388	0.32	0.47	0.00	1.00
Amount of Donation	81,380	35.16	113.51	0.00	6,000.00
ln(Amount of Donation+1)	81,380	1.30	1.98	0.00	8.70
Equivalence-adjusted Gini Coefficient Across Regions	81,388	0.35	0.03	0.29	0.48
ln(Equivalence-adjusted Average Household Consumption of Bottom 5% Consumption Group +1)	81,388	5.91	0.23	5.03	6.57
Donor’s Income Level / Average Consumption of Bottom 5% Consumption Group	81,388	5.57	3.88	-0.03	40.71
Equivalence-adjusted Financial Assets	80,755	1,260.27	3,586.33	0.00	228,034.00
ln(Equivalence-adjusted Financial Assets+1)	80,755	4.36	3.39	0.00	12.34
Equivalence-adjusted Current-Term Income	81,363	2044.13	1381.61	0.00	14,231.65
ln(Equivalence-adjusted Current-Term Income+1)	81,363	7.31	1.06	0.00	9.56
Equivalence-adjusted Next-Term Income	72,719	2,199.23	1907.56	0.00	84,447.10
ln(Equivalence-adjusted Next-Term Income+1)	72,719	7.39	0.98	0.00	11.34
Tax price of donations	81,388	0.92	0.04	0.59	1.00
Age	81,388	53.09	15.40	15.00	100.00
(Age^2)/1000	81,388	3.06	1.71	0.23	10.00
Married	81,388	0.69	0.46	0.00	1.00
Female	81,388	0.23	0.42	0.00	1.00
Urban Dweller	81,388	0.47	0.50	0.00	1.00
Family Size	81,388	2.84	1.32	1.00	10.00
No. of Family Member under Age 15	81,388	0.51	0.83	0.00	5.00
No. of Family Member over Age 60	81,388	0.56	0.77	0.00	4.00
Education (Years)	81,306	11.24	4.52	0.00	26.00
Wage Worker	81,388	0.71	0.45	0.00	1.00
Self-employed	81,388	0.23	0.42	0.00	1.00
Buddhism	81,388	0.21	0.41	0.00	1.00
Christianity	81,388	0.18	0.39	0.00	1.00
Catholicism	81,388	0.06	0.24	0.00	1.00
Other Religions	81,388	0.01	0.10	0.00	1.00
Drinking	81,230	0.67	0.47	0.00	1.00
Smoking	81,230	0.38	0.48	0.00	1.00
Health Condition (5-point scale)	81,376	3.23	0.80	1.00	5.00
BMI index	67,044	23.21	2.69	12.86	63.55
(BMI index^2)/1,000	67,044	5.46	1.30	1.65	40.39

We use the dependent variable of ‘Engaged in Donation’ in the selection equation and ln (Amount of donation+1) as the dependent variable in the estimation equation. To ensure mathematical validity, we log-transform the dependent variable as ln(donation amount + 1) rather than ln(donation amount). This adjustment is crucial because the logarithm of zero is undefined, and donation amounts can potentially be zero. Similarly, any variables related to the amount also undergo this transformation to maintain mathematical consistency. The variable ’Engaged in Donation’ in the selection equation is set to 1 if a donation is made, and 0 otherwise, with an average of about 32% of all households engaging in donations.

The independent variables are classified into four types. The first set of variables are supposed to affect the equation determining the amount of donation. As stated in theoretical discussion above, we include the variables representing a donor’s relative income and consumption, ln(equivalence-adjusted financial assets+1), ln(equivalence-adjusted current-term income+1), ln(equivalence-adjusted next-term income+1), and the tax price of donations. Moreover, the tax price of donations is used as an independent variable determining the amount of donation. Because a part of donation is returned to the donator in the form of tax deductions, the tax price of donations differs depending on a donor’s income, and the number of household members, etc. Hence, the tax price of donations here means how much a donor can give by donating KRW 1, which is obtained by the following equation: 1- (calculated tax amount/tax base).

The second group of independent variables are concerning individual and household characteristics. In our dataset, marital status is coded as 1 for married individuals and 0 for unmarried individuals (including divorced and single). Similarly, the variable "female" indicates households where the head of the household is female. Variables for individual characteristics include the age, marital status, gender, region of residence, education, employment status, etc., whereas household characteristics refer to the number of members in the household, and the number of household members under age 15 or over 60.

The third are independent variables regarding religion, while the fourth is individual health condition where respondents are asked to rate their health on a scale of 1–5. Also included is the body mass index measured by the value of weight divided by the square of height. In general, the BMI value under 20 is regarded as thinness, and those over 25 are classified into obese class. Table 3 offers a regional breakdown of the equivalence-adjusted Gini coefficient and the equivalence-adjusted average consumption for the bottom 5% of consumers.

**Table 3:** Equivalence-adjusted gini coefficient and equivalence-adjusted average consumption of bottom 5% consumption group across region.

Region	Equivalence-adjusted Gini Coefficient	Equivalence-adjusted Average Consumption of Bottom 5% Consumption Group(10 thousand won/year)
Seoul	0.335	520.1
Busan	0.333	491.2
Daegu	0.340	415.8
Incheon	0.296	568.7
Gwangju	0.355	359.1
Daejeon	0.313	400.9
Ulsan	0.300	712.9
Gyeonggi-do	0.305	481.1
Gangwon-do	0.350	386.6
Chungcheongbuk-do	0.302	467.5
Chungcheongnam-do	0.359	344.9
Jeollabuk-do	0.348	287.6
Jeollanam-do	0.370	316.8
Gyeongsangbuk-do	0.340	360.3
Gyeongsangnam-do	0.298	490.7

Building on existing models, this study offers a fresh perspective on donation behavior by employing a novel utility function. Unlike traditional approaches, this function explicitly factors in the difference in consumption between donors and recipients, addressing a key issue raised in our research question.

The theoretical model adopted in this study analyzes the impact of several variables on donation by using a utility function taking a differentiated form from theory in previous literature. Our utility function reflects the differences in consumption between a donor and a recipient, mentioned in the research question in the previous section.


$$U_{it}=U(C_{it})+\gamma_{i}\{U(C_{jt},D_{it})-U(C_{jt})\},$$ (1)

We define a utility function as (1), where i denotes an individual economic actor, j is an individual economic actor who is receiving donation from i, and t means the point of time. U refers to total utility that is determined by *U*(*C*_*it*_), utility arising from a donor’s consumption, and $\gamma_{i}\{U(C_{jt},D_{it})-U(C_{jt})\}$, a function of a recipient’s net utility increase arising from donation (*D*_*it*_). The path via which donation increases utility in Eq (1) is as follows. Donation increases a recipient’s utility, and a certain proportion (*γ*_*i*_) of the net increase of utility again increases a donor’s utility.

To simplify the maximization of the utility function such as Eq (1), we apply a log utility function to consumption and the endowment one-period model assuming only two parties to finally get the utility maximization problem such as Eqs (2), (3), and (3)’ as follows.


$${Max\;U}_{i}=\ln\;C_{i}+\gamma_{i}\;\ln\left(C_{j}+D_{i}\right)-\gamma_{i}ln(C_{j}),\;where\;C_{i}\geq C_{j},{0\leq \gamma }_{i}\leq 1$$ (2)

$$s.t.\;Y_{i}-C_{i}-D_{i}=0,\;where\;\{Y_{i},C_{j}\}\;are\;exogenous$$ (3)

$$⟹D_{i}=Y_{i}-C_{i},$$ (3)’

To solve the utility maximization problem, we substitute the Eq (3)’ into Eq (2), and finally obtain Eq (4).


$${Max\;U}_{i}=\ln\;C_{i}+\gamma_{i}\;\ln\left(C_{j}+Y_{i}-C_{i}\right)-\gamma_{i}ln\;(C_{j}),$$ (4)

By solving Eq (4) using FOC, we have a solution to private consumption (*C*_*i*_) as below in Eq (5).


$$FOC:\;\frac{{\partial U}_{i}}{{\partial C}_{i}}=\frac{1}{C_{i}}+\gamma_{i}\left(\frac{-1}{Y_{i}-C_{i}+C_{j}}\right)=0$$

$$\frac{1}{C_{i}}=\frac{\gamma_{i}}{Y_{i}-C_{i}+C_{j}}⟹\gamma_{i}C_{i}=Y_{i}-C_{i}+C_{j}⟹(1+\gamma_{i})C_{i}=Y_{i}+C_{j}⟹C_{i}=\frac{Y_{i}+C_{j}}{{1+\gamma }_{i}},$$ (5)

Finally, we substitute Eq (5), the solution to private consumption (*C*_*i*_) into Eq (3)’ to obtain a solution to individual donation (*D*_*i*_) as shown in Eq (6).


$$D_{i}=Y_{i}-C_{i}⟹D_{i}=Y_{i}-\frac{Y_{i}+C_{j}}{1+\gamma_{i}}⟹D_{i}=\frac{\gamma_{i}Y_{i}}{1+\gamma_{i}}-\frac{C_{j}}{1+\gamma_{i}}$$ (6)

In Eq (6), an amount of donation could be expressed as a functional relationship between an individual economic actor’s income and a recipient’s consumption. An individual economic actor’s donation that is determined by the utility maximization problem is a function of consumption by a recipient where an actor’s donation decreases as a recipient’s consumption rises. This occurs because the incentive for donation falls when the donor-recipient gap in marginal utility decreases.

As donations are zero when there are no donations, the donation model is similar to a left-censored model. However, if an actor first decides whether or not to donate, and then chooses the amount of donation, this can lead to sample selection bias. To address this issue, most previous studies have used the Tobit model or the Heckman two-stage model. This study also adopts the double-hurdle model in addition to the Heckman two-stage model. Our analysis model can be expressed in Eqs (7) through (9). Basically, the amount of donation becomes zero when there is no donation, which is similar to the left-censored model. However, if an actor first decides whether to donate or not, and then chooses the amount of donation, this gives rise to sample selection bias. Taking into account that, most previous studies turned to the Tobit model as well as the Heckman two-stage model. On top of the Heckman two-stage model, this study also adopts the double-hurdle model for carrying out analysis. Simply put, out analysis model could be expressed as Eq (7) through (9).


$$s_{it}^{*}=w_{it}^{\prime }\alpha +\varepsilon_{it},s_{it}=1[s_{it}^{*}>0],$$ (7)

$$D_{it}^{*}=x_{it}^{\prime }\beta +u_{it},D_{it}=D_{it}^{*}1[D_{it}^{*}>0]=Max(D_{it}^{*},0)$$ (8)

$$x_{it}=\{\ln\;C_{it},\ln\;C_{it+1},\ln\;C_{jt},\ln B_{it},p\},w_{it}=\{\mathrm{personal}\;\mathrm{characteristic}\;\mathrm{variables}\}$$

$$(s_{it},x_{it},w_{it},D_{it})\;\mathrm{are}\;\mathrm{observed},$$ (9)

Eq (7) is regarding selection, whereas Eq (8) is for deciding the amount of donation. Eq (9) refers to independent variables. *x*_*it*_ means independent variables related to a decision on how much an individual donates. As explored in the theoretical ground, *x*_*it*_ variables are a donator’s income at the current and subsequent terms, a recipient’s income, and the tax price of donations. *w*_*it*_ refer to independent variables that explain sample selection, including personal elements including age, education, family composition, place of residence, employment status, religion, physical characteristics, etc.

This study uses two latent variables, $s_{it}^{*}$ and $D_{it}^{*}$ to represent donation behavior. Because there are two latent variables, we use a double-hurdle approach for our estimation model. We link our theoretical model with the Probit model to arrive at Eq (10), and then linearize it to obtain Eq (11). Because there two latent variables, we select the double-hurdle approach for our estimation model. By linking our theoretical model with the empirical one, we arrive at Eq (10), and Eq (11) after linear approximation. First of all, in theory, *γ*_*it*_ determines whether an individual intends to donate or not. Although an individual has extremely high income, (s)he has no incentive for donation if *γ*_*it*_ turns zero. What this means in our estimation is that $s_{it}^{*}$ in Eq (7) serves a similar function to *γ*_*it*_, making *D*_*it*_ become zero. In the case *γ*_*it*_ is not zero, *D*_*it*_ turns into zero if $D_{it}^{*}$ becomes equal to or lower than zero. This gives rise to two types of hurdles, which makes it reasonable for us to turn to the Heckman model and the double-hurdle model for our estimation.


$$s_{it}^{*}=\gamma_{it},D_{it}^{*}=\frac{\gamma_{it}}{p_{it}(1+\gamma_{it})}({\bar{Y}}_{i}+r{\bar{B}}_{i})-\frac{C_{jt}}{1+\gamma_{it}},$$ (10)

$$D_{it}=\beta_{1}+\beta_{2}\;{\ln\;C}_{it}+\beta_{3}\;{\ln\;Y}_{it}+\beta_{4}\;{\ln\;Y}_{it+1}+\beta_{5}\;{\ln\;B}_{it}+\beta_{6}p_{it}+\varepsilon_{it},$$ (11)

This equation is a regression model that attempts to explain the amount of money donated by individual *i* at time *t* as a function of such independent variables as individual consumption, individual income, individual expected income, individual wealth, and the proxy for individual altruism. The coefficients measure the strength of the relationship between each independent variable and the amount of money donated. The error term *ε*_*it*_ captures all other factors that may influence the amount of money donated that are not included in the model.

Finally, the parameters to be estimated are *α*, *β*, and *σ*_*u*_, with the log-likelihood function of our double-hurdle model as shown in Eq (12) below.


$$\sum_{i=1}^{N}\sum_{t=1}^{T}\left[(1-s_{it})\ln\left\{1-\Phi (w_{it}^{\prime }\alpha )\Phi (x_{it}\prime \frac{\beta }{\sigma_{u}})\right\}+s_{it}\;\ln\left\{\Phi (w_{it}^{\prime }\alpha )\frac{1}{\sigma_{u}}\Phi (x_{it}\prime \frac{\beta }{\sigma_{u}})\right\}\right]$$ (12)

## Results

Table 4 presents our findings obtained using two models: The Heckman two-stage and the double-hurdle. Interestingly, for the decision to donate, both models yield identical results regardless of the included variables. However, when it comes to the amount donated, the story differs, and only the results from the Heckman model are reported in the left column.

**Table 4:** Empirical results.

	Heckman Two-Stage				Double hurdle			
	Donation Decision	Amount of Donations			Donation Decision	Amount of Donations		
Constant Term	-5.124*** (0.239)	6.107*** (0.312)			-5.087*** (0.244)	6.073*** (0.311)		
Equivalence-adjusted Gini Coefficient Across Regions		1.962*** (0.299)				1.959*** (0.299)		
ln(Equivalence-adjusted Average Consumption of Bottom 5% Consumption Group +1)			-0.166*** (0.033)				-0.169*** (0.033)	
Donor’s Equivalence-adjusted Household Income / Equivalence-adjusted Average Household Consumption of Bottom 5% Consumption Group				0.029*** (0.003)				0.028*** (0.003)
ln(Equivalence-adjusted Financial Assets+1)		0.009*** (0.002)	0.009*** (0.002)	0.007*** (0.002)		0.009*** (0.002)	0.009*** (0.002)	0.006*** (0.002)
ln(Equivalence-adjusted Current-Term Income+1)		0.064*** (0.011)	0.062*** (0.011)	0.033*** (0.012)		0.066*** (0.012)	0.064*** (0.012)	0.035*** (0.012)
ln(Equivalence-adjusted Next-Term Income+1)		0.157*** (0.010)	0.157*** (0.010)	0.144*** (0.010)		0.159*** (0.010)	0.158*** (0.010)	0.146*** (0.010)
Tax Price of Donations		-3.928*** (0.241)	-4.002*** (0.241)	-2.385*** (0.290)		-3.947*** (0.237)	-4.023*** (0.237)	-2.486*** (0.279)
Age	0.056*** (0.003)				0.055*** (0.003)			
(Age^2)/1000	-0.382*** (0.029)				-0.357*** (0.030)			
Marital Status	0.330*** (0.020)				0.321*** (0.021)			
Female	0.207*** (0.021)				0.219*** (0.022)			
Urban Dweller	-0.055*** (0.011)				-0.058*** (0.012)			
Family Size	0.048*** (0.007)				0.043*** (0.007)			
No. of Family Member under Age 15	0.012 (0.009)				0.015 (0.010)			
No. of Family Member over Age 60	0.070*** (0.011)				0.059*** (0.012)			
Education (Years)	0.043*** (0.002)				0.048*** (0.002)			
Wage Worker	0.146*** (0.017)				0.163*** (0.017)			
Self-employed	-0.075*** (0.015)				-0.084*** (0.015)			
Buddhism	0.322*** (0.015)				0.304*** (0.015)			
Christianity	1.400*** (0.015)				1.387*** (0.016)			
Catholicism	1.048*** (0.022)				1.037*** (0.023)			
Other Religions	0.780*** (0.049)				0.744*** (0.050)			
Drinking	-0.100*** (0.014)				-0.092*** (0.014)			
Smoking	-0.113*** (0.013)				-0.118*** (0.013)			
Health Condition (5-point scale)	0.080*** (0.008)				0.076*** (0.008)			
BMI index	0.086*** (0.019)				0.084*** (0.019)			
(BMI index^2)/1,000	-0.156*** (0.039)				-0.151*** (0.040)			
Number of Samples	64,474	64,474	64,474	64,474	60,162	60,162	60,162	60,162

Note: Numbers in parentheses are standard deviations. ***, **, and * means the significance level of 1%, 5%, and 10%, respectively.

Our study confirms and expands upon the established link between individual and household characteristics and donation decisions. Examining this relationship in the Korean context, we not only replicate the findings of previous research but also uncover unique patterns and motivations specific to this population. This reinforces the consistency of these factors while adding valuable context and nuance to the existing knowledge base. The sole exception emerged with the equivalence-adjusted average household consumption of the bottom 5% of households. Our first key finding is the positive link between income inequality (measured by the Gini coefficient) and individual donations. It is important to see how a higher Gini coefficient might affect the likelihood of individuals making the initial decision to donate. We may consider factors like increased awareness of inequality, potential for higher returns on donations in high-inequality settings, or moral motivations driven by concerns about fairness. Also, we analyze how the magnitude of the Gini coefficient could influence the amount individuals choose to donate. It is likely whether a larger coefficient might trigger larger donations due to feelings of obligation or guilt, or if potential diminishing returns in situations of extreme inequality could lead to lower contributions. Donors from regions with higher inequality tend to give more generously, supporting our theoretical prediction that relative income drives charitable giving. Reassuringly, our analysis of individual and household characteristics influencing donation decisions mirrors the findings of most previous studies. This agreement bolsters the validity of existing theoretical frameworks and demonstrates consistency between past and present research. Several factors significantly influence individuals’ decisions to donate at the 1% level. Married individuals, women, those with higher education, and religious affiliation generally give more. Age also plays a role, with older adults and those with health conditions (but not BMI) more likely to contribute. Meanwhile, living in urban areas, being self-employed, or engaging in unhealthy habits like drinking and smoking tend to discourage giving. Our analysis of both donation decisions and amounts largely confirms existing research. Table 5 details the close alignment between our findings and theoretical predictions, bolstering the validity of past models and enriching our understanding of charitable giving.

**Table 5:** Comparisons of predictions and empirical results.

Step 1: Decide to Donate	Predictions	Empirical Results
Age	Positive correlation	age (+), age^2^(-)
	Concave in quadratic function	Concave in quadratic function
Marital Status	Positive correlation between being married and donation	Positive correlation confirmed between being married and donation
Female	Females more likely to donate	Females more likely to donate
	Correlation not evident in many studies	
Education	Positive correlation evidently confirmed	Positive correlation evidently confirmed
Family Composition	Positive correlation between number of children and donation	Positive correlation between number of the elderly and donation
	Correlation not evident in many studies	
		Number of children not clearly related to donation
Religion	Positive correlation between being religious and donation	Significantly positive correlation in the order of Christianity, Catholicism, other religions, and Buddhism
Step 2: Decide the Amount of Donation	Predictions	Our Empirical Results
Income Inequality (Regional Gini Coefficient)	(+)	(+)
	Deepening inequality (C_C_jt_) helps increase donation	Positive effect of deepening income inequality on the amount of donation
Relative Income (Donor’s Equivalence-adjusted Household Income / Equivalence-adjusted Average Household Consumption of the Bottom 5%)	(+)	(+)
	Increased relative income (C/C_jt_) helps increase the amount of donation	Positive effect of increased relative income on the amount of donation
Financial Assets	(+)	(+)
	Higher financial assets increasing the amount of donation	Higher financial assets increasing the amount of donation
Current Income	(+)	(+)
	Higher current income increasing the amount of donation	Higher current income increasing the amount of donation
Next-term Income	(+)	(+)
	Higher future income increasing the amount of donation	Higher future income increasing the amount of donation
Tax Price of Donations	(-)	(-)
	Higher tax price of donation lowering the amount of donation	Higher tax price of donation lowering the amount of donation

## Conclusions and limitations

This study delves into the factors influencing individual generosity, specifically exploring the role of relative income. We track changes in donations and personal characteristics over time using panel data, allowing us to build a robust model that supports our theoretical framework. This framework incorporates a broader definition of utility compared to past studies, accounting for how differences in income between donors and recipients impact their satisfaction. Our research reveals that how much you spend compared to others (relative consumption) is the main driver of your charitable giving, even more than your current income. This aligns with our theoretical model, which predicts that factors like your relative wealth and future income prospects also play a role, along with the cost of giving itself. Notably, we also find that donors tend to give more when their contribution makes a big difference for the recipient, highlighting the role of altruistic motivation. Breaking new ground, our study offers a more complete model exploring how individuals decide how much to spend, donate, and save. Unlike past research, we consider not just the donor’s own situation but also the recipient’s economic hardships and the broader societal trends in consumer spending. This reveals that helping those struggling financially is a top priority for most donors, making the recipient’s consumption level a key factor in whether a donation is made.

To understand how people decide how much to spend, donate, and save, we create a new model that considers both their own happiness and the impact their donations have on others. This model takes into account how much better off someone is after receiving a donation, helping us explain how generosity works. Past research on donation behavior used models like Tobit and Heckman two-stage, considering factors like income, wealth, and religion. While they found these factors to positively influence giving, their models might have missed some important information. Our study takes a step forward by employing a double-hurdle model, tackling potential biases in both deciding whether to donate and how much to give. This allows us to provide a more accurate picture of what drives generosity. Unlike previous research, our study considers how your spending power compared to others (relative income) influences your desire to donate. This new perspective lets us pinpoint the factors that truly drive charitable giving in both theory and practice. To ensure accurate results, we use sophisticated tools like the Heckman two-stage approach and the double-hurdle model, which account for potential biases and limitations in the data.

While our study sheds light on factors influencing donation decisions, it’s important to acknowledge some limitations. One concerns the use of the regional Gini coefficient to capture recipient circumstances. This measure reflects overall regional inequality, but it doesn’t consider that recipients might compete for resources with individuals from other regions, potentially affecting its accuracy in representing their specific situations. Another study limitation concerns using the ratio of a donor’s income to the bottom 5%’s average as a proxy for recipient need. This measure raises concerns because it implicitly assumes all recipients belong to the lowest income bracket, potentially misrepresenting the diversity of their socioeconomic situations. The measure used in this study overlooks several points. First, many recipients outside the bottom 5% may struggle financially. Second, individual needs vary widely, and this measure doesn’t capture that diversity. Finally, cost-of-living differences across regions further complicate its accuracy. Future research should explore how much of a donation in one region actually stays within that region and benefits local recipients. Our study utilizes a comprehensive annual survey, but capturing donations to rural Korean areas remains a challenge. This is because such donations often flow through informal channels like social networks, making them difficult to track. Understanding the true extent of financial aid reaching rural communities requires addressing these data collection hurdles. Understanding how much of a donation stays within the intended region and benefits local recipients is crucial. Further research on this question can enhance the effectiveness of charitable giving, ensuring support reaches those who need it most.

This study examines how donations impact low-income groups, even though the available data only captures the total amount donated, not its specific purpose. While donations can support various causes like nature restoration and disease eradication, we assume them to be directed towards lower-income individuals due to the data limitations. Ideally, future research could benefit from more detailed data collection on donation usage patterns. However, existing evidence suggests a significant portion of Korean donations support domestic and local communities, as observed by Song (2016) [7]’s finding that 65.5% of donors contribute to such activities.

## Supporting information

S1 Data (CSV)
